# Attention or instruction: Do sustained attentional abilities really differ between high and low hypnotisable persons?

**DOI:** 10.1007/s00426-017-0850-1

**Published:** 2017-03-07

**Authors:** Jean-Rémy Martin, Jérôme Sackur, Zoltan Dienes

**Affiliations:** 1 School of Psychology, University of Sussex, Brighton, UK; 2 École des Hautes Études en Sciences Sociales (EHESS), PSL Research University, Département d’études cognitives, Laboratoire de Sciences Cognitives et Psycholinguistique (CNRS/ENS/EHESS), Paris, France; 3 École Polytechnique, Palaiseau, France; 4 Sackler Centre for Consciousness Science, University of Sussex, Brighton, UK

## Abstract

Previous research has suggested that highly hypnotisable participants (‘highs’) are more sensitive to the bistability of ambiguous figures—as evidenced by reporting more perspective changes of a Necker cube—than low hypnotisable participants (‘lows’). This finding has been interpreted as supporting the hypothesis that highs have more efficient sustained attentional abilities than lows. However, the higher report of perspective changes in highs in comparison to lows may reflect the implementation of different expectation-based strategies as a result of differently constructed demand characteristics according to one’s level of hypnotisability. Highs, but not lows, might interpret an instruction to report perspective changes as an instruction to report many changes. Using a Necker cube as our bistable stimulus, we manipulated demand characteristics by giving specific information to participants of different hypnotisability levels. Participants were told that previous research has shown that people with similar hypnotisability as theirs were either very good at switching or maintaining perspective versus no information. Our results show that highs, but neither lows nor mediums, were strongly influenced by the given information. However, highs were not better at maintaining the same perspective than participants with lower hypnotisability. Taken together, these findings favour the view that the higher sensitivity of highs in comparison to lows to the bistability of ambiguous figures reflect the implementation of different strategies.

**Electronic supplementary material:**

The online version of this article (doi:10.1007/s00426-017-0850-1) contains supplementary material, which is available to authorized users.

## Introduction

Within the field of hypnosis, it is widely acknowledged that people are not equally responsive to hypnotic suggestions (suggestions for altered experiences of reality or volition) (Heap, Brown, & Oakley 2004; Hilgard 1965; Kallio & Ihamuotila 1999; Laurence, Beaulieu-Prévost, & Du Chéné 2008; Perry, Nadon, & Button 1992; Shor & Orne 1962; Weitzenhoffer & Hilgard 1962; but see; Barber 1969; Spanos 1986). Although it is largely believed by researchers that differences in hypnotisability must be reflected in participants’ other traits (i.e., from outside the hypnotic context), attempts to determine potential different cognitive profiles between highs and lows rarely find replicated correlates that predict with better than *r* = 0.2, when tested in unrelated contexts (Council, Kirsch, & Hafner 1986; Heap, Brown, & Oakley 2004; Laurence, Beaulieu-Prévost, & Du Chéné 2008). In addition, there is no firm genetic, physiological, behavioural or phenomenological marker differentiating highs from lows. In sum, why some people are more responsive to hypnotic suggestions than others is still an unresolved issue.

One of the more promising avenues of research has been the hypothesis that highs exhibit more efficient executive control than lows, evidenced by greater sustained and selective attentional abilities (Crawford 1991, 1994; Crawford, Brown, & Moon 1993; for a recent review about the role of frontal executive functions in hypnosis, see; Parris in press). However, results for different baseline performances—from outside the hypnotic context—according to participants’ hypnotisability level in various executive and attentional dimensions are mixed, with studies reporting no significant behavioural differences (Cojan, Piguet, & Vuilleumier 2015; Dienes et al. 2009; Egner, Jamieson, & Gruzelier 2005; Iani, Ricci, Gherri, & Rubichi 2006; Iani, Ricci, Baroni, & Rubichi 2009; Raz, Fan, & Posner 2005; Varga, Németh, & Szekely 2011) but, importantly, other studies showing significant differences in either direction (Crawford et al. 1993; Dixon, Brunet, & Laurence 1990; Dixon & Laurence 1992; Farvolden & Woody 2004; Miller, Hennessy, & Leibowitz 1973; Miller 1975; Rubichi, Ricci, Padovani, & Scaglietti 2005; Wallace 1986; Wallace & Garrett 1973; Wallace, Garrett, & Anstadt 1974; Wallace, Knight, & Garrett 1976). Recently, a new layer of complexity has been added to this already tangled issue, as it has been shown (Cojan et al. 2015; Lifshitz & Raz 2015) that similar behavioural levels of Stroop or Stroop-like interference between highs and lows were accompanied by different patterns of neural activity. Highs and lows may have different cognitive styles or different context-dependent strategies.

Potential attentional differences between highs and lows have been investigated by means of different experimental procedures, especially by means of conflicting (e.g., Stroop), distracting or priming paradigms (Cojan, Piguet, & Vuilleumier 2015; Dienes et al. 2009; Iani et al. 2009; Raz et al. 2005; Varga et al. 2011) and, finally, by means of specific visual illusions. Some evidence suggest that highs and mediums are more sensitive than lows to the Ponzo illusion, that highs report more changes in direction of autokinetic movement (illusory movement of a light in a dark room) and more reversals of a Necker cube than lows (Crawford et al. 1993; Miller et al. 1973; Miller 1975; Wallace & Garrett 1973; Wallace et al. 1974, 1976; Wallace 1986; but see; Jamieson & Sheehan 2002). Previous research advocated that responsiveness to bistable figures depends on focusing attention towards the relevant and salient cues while filtering (disattending) irrelevant cues (Power & Day 1973). In this regard, Crawford and colleagues (1993) interpret the higher sensitivity of highs in comparison to lows to bistable figures and visual illusions as reflecting different attentional abilities; with highs showing more efficient sustained attentional and disattentional abilities than lows. In other words, highs may report a higher rate of perspective switches in a Necker cube because of a higher ability to focus on the salient cues and to disattend the non-salient ones.

However, the higher sensitivity of highs to bistable figures or visual illusions might reflect expectation-based strategy differences rather than baseline attentional differences (Dienes et al. 2009). We have to know what participants are trying to achieve during the task otherwise results are difficult to interpret. If the instruction consists in asking participants to report every perspective change, as was the case in previous studies comparing highs and lows on the perception of bistable figures (Crawford et al. 1993; Wallace 1986; Wallace et al. 1976), the higher rate of switches by highs might reflect the implementation of specific strategies in order to fulfil what they thought to be a “good high” in this context (Orne 1959, 1969; Spanos 1986; for recent instances of the effect of demand characteristics in different perceptual phenomena and new methods to unveil them, see; Firestone 2013; Firestone & Scholl 2014; Martin, Sackur, Anlló, Naish, & Dienes 2016). That is, they might have inferred that the experimenter expected them to see many perspective switches, while lows are simply neutral about the kind of switch rate expected by the experimenter. To be clear, we are not arguing that highs may be better than lows or mediums in interpreting demand characteristics, but that different demand characteristics are inferred given one’s level of hypnotic suggestibility. In addition, or alternatively, it might also be argued that lows might have been less motivated than highs in doing the task, reporting less switches than highs.

Here, we shall assay the weight of demand characteristics in behaviours that have been attributed to differential attentional abilities across hynotisability levels. Namely, we tested whether when asked to report perceptual switches of a bistable figure, participants would differentially adapt their performance to specific information according to their level of hypnotisability.

We tested this hypothesis using a Necker cube as the bistable stimulus. Using a design close to the one of Crawford et al. (1993), we tested three groups of participants; highs, mediums and lows, thus spanning the whole spectrum of hypnotisability levels. Participants had to report every perspective change of a Necker cube. In the first block—Neutral Block—of trials, no specific information was given to participants. In the second block—Test Block—, participants were either informed that previous research had demonstrated that people with their specific level of hypnotisability had been shown to be able to change perspective easily (Switch Condition) or to maintain the same perspective easily (Maintain Condition) and that we would like to test this hypothesis with a Necker cube. Our inclusion of the group of mediums enables us to test whether any difference in the rate of perspective switches was due to highs or lows showing extreme behaviour.

## Method

### Participants

Participants who took part in this experiment had been screened for hypnotisability with the French version of the Harvard Group Scale of Hypnotic Susceptibility, Form A (Shor & Orne 1962; Anlló, Becchio & Sackur 2017). This scale consists in a relaxation-based induction phase followed by 12 suggestions, encompassing cognitive suggestions (e.g., hallucination), motor suggestions (e.g. hands moving together) and challenge suggestions (to not succeed at an action e.g. to not be able to bend the arm because of arm rigidity). Subjects’ score is determined by the number of suggestions they pass according to specific criteria. As an illustration, the magnetic hands suggestion is phrased as followed:

“Now I want you to imagine a force attracting your hands toward each other, pulling them together. As you think of this force pulling your hands together, they will move together, slowly at first, but they will move closer together, closer and closer together as though a force were acting them… moving… moving… closer… closer….” (Shor & Orne 1962, p. 9).

We recruited 21 highly hypnotisable participants (highs) scoring 9–12 (*M* = 9.6, SD 0.8; 12 females; mean age 24.3, SD 3.6), 24 moderately hypnotisable participants (mediums) scoring 5–8 (*M* = 6.2, SD 1.2; 18 females; mean age 25.3, SD 4.3) and 23 low hypnotisable participants (lows) scoring 0–4 (*M* = 2.6, SD 1.2; 12 females; mean age 25.6, SD 4.5). Each subject was paid 5 € for participation, the whole experiment lasting approximately 30 min. Participants had normal or corrected-to-normal vision.

Written informed consent was obtained from each participant and the experiment was conducted in agreement with the Declaration of Helsinki (2008) and approved by the Ethics Committee of the Université Paris Descartes (Paris 5).

### Experimental setup and apparatus

The experiment was conducted in a quiet experimental room. Stimuli were delivered by a MacBook Pro, processor 2.53 GHz, Intel Core i5. All stimuli were displayed using Matlab (MathWorks Inc R 2009b) with the Psychophysics Toolbox (Brainard 1997).

### Stimuli and experimental design

**Fig. 1:**
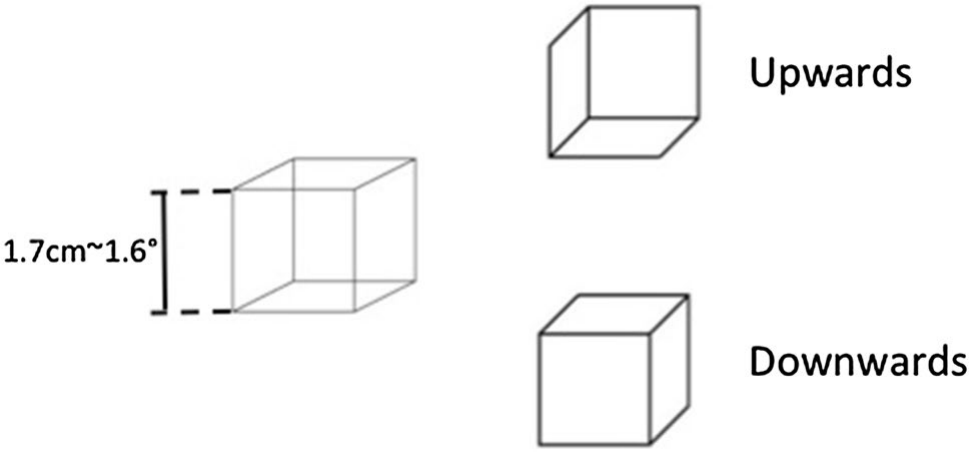
Stimulus and procedure. In the instruction phase participants were shown the ambiguous cube (*left cube*) accompanied by two non-ambiguous cubes (*right bold cubes*) showing the two possible alternatives the ambiguous cube could alternate between. Participants were described the perspective shown by the *bold cube on the top* as the upwards perspective and the *bold cube on the bottom* as the downwards perspective. Participants were instructed to press the key with an upwards arrow drawn on it (*j* key) when the cube switched from the downwards to the upwards perspective and the key with a downwards arrow drawn on it (*f* key) when the cube switched from the upwards to the downwards perspective

Participants were seated at about 60 cm from the screen. A Necker cube (edge size 1.7 cm/~1.6°) was displayed against a light-grey background at the centre of the screen during periods of 60 s. During these periods, participants had to report each time they saw a change in cube perspective by means of key presses. As training, before the first period, participants were shown the ambiguous Necker cube accompagnied by two non-ambiguous cubes, illustrating the two possible interpretations of the ambiguous cube (see Fig. 1). Participants were asked to tell the experimenter once they observed that the ambiguous cube could indeed switch back and forth between the two perspectives represented by the non-ambiguous cubes.

The experiment comprised two blocks composed of five 60 s periods each. There was a short break of 5 s between each consecutive 60 s period. The first block (Neutral Block) was presented to participants as a training block. Instructions were as close as possible to Crawford et al. (1993):

#### Training

The cube will be displayed for 60 s at a time. Press the key with an upwards arrow drawn on it [corresponding to the *j* key] when you see the cube changing direction upwards, press the key with a downwards arrow drawn on it [corresponding to the *f* key] when you see the cube changing direction downwards. When you are looking at the cube, look at it as you normally would. Do not blink excessively [Translated from the French version given to participants].

The second block (Test Block), presented as the test block to participants, was preceded by specific information. According to the condition participants were (randomly) assigned to—Switch Condition (SC) or Maintain Condition (MC)—the information specified that previous research had shown that they were able to shift perspective easily or to maintain the same perspective easily, respectively:

#### Second phase

We know that people like you with high (for mediums: *some*) hypnotic abilities (for lows: *that are resistant to hypnosis*) have a great ability to change (in the SC) (*to maintain the same*, in the MC) perspective. We would like to test this hypothesis with the cube. As before, when you are looking at the cube, look at it as you normally would. Do not blink excessively [Translated from the French version given to participants].

Because the nature of the information given to participants depended on their hypnotisability level, at the very beginning of the experiment (i.e., before Neutral Block and its instruction phase) participants were reminded what their level of hypnotisability was:

We recruited you for this experiment because you have shown high (for mediums: *some*) hypnotic abilities (for lows: *showed resistance to hypnosis*) during screening [Translated from the French version given to participants].

## Results

Two participants were excluded because debriefing showed that they did not understand the task. One participant was excluded because 64% of her/his key presses were identical key responses, suggesting again a misunderstanding about the task. In total, we thus rejected two participants from the group of highs and one from the group of lows. Episodes separated by repeated key presses were then conjoined. Next, we rejected as outliers episodes shorter than 600 ms (1.8%) and more than three standard deviations above each participant median duration (2.2%).

### Mean frequency of perspective switches according to groups and conditions

**Fig. 2:**
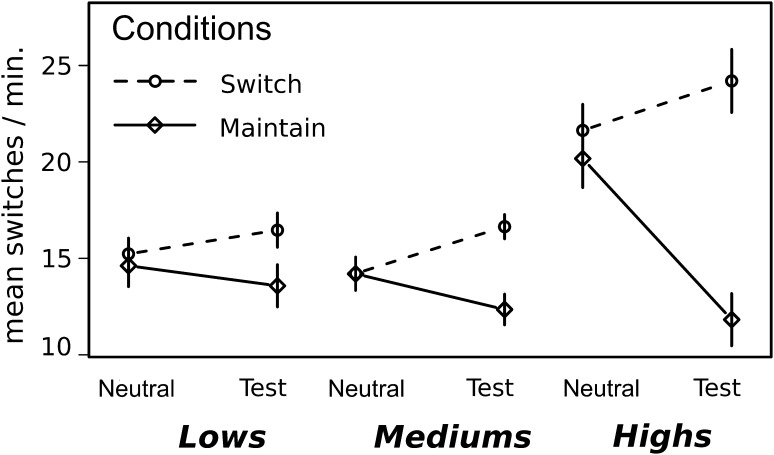
Mean frequency of perspective changes. Graph shows the mean frequency of switches per minute for Neutral and Test Block, for every group (lows, mediums and highs) and, finally, for the Switch (*dashed line*) and Maintain (*continuous line*) condition

Figure 2 shows the mean frequency of perspective switches according to groups and conditions. Mean number of switches per minute was numerically higher in highs (*M* = 21, SE = 2.2) than in lows (*M* = 15, SE = 1.3) and mediums (*M* = 14.2, SE = 1.1) in the Neutral Block. As for Test Block, in the Switch Condition, highs (*M* = 24.2, SE = 3.6) switched numerically more than lows (*M* = 16.5, SE = 1.7) and mediums (*M* = 16.6, SE = 1.1). Finally, in the Maintain Condition, while every group seemed to switch less than in the Switch Condition, no group markedly differentiated from one another as shown by their mean frequency of switches; highs (*M* = 11.8, SE = 2.9), lows (*M* = 13.6, SE = 2.5), mediums (*M* = 12.3, SE = 1.3).

### Bayes factors

In order to evaluate the strength of evidence for the alternative hypotheses H1 versus H0 (Morey, Romeijn, & Rouder 2016), we report Bayes Factors, *B*, for the relevant tests with one degree of freedom. Following Jeffrey (1939), we consider that a *B* above 3 indicates “substantial evidence” for H1 over H0 and, by symmetry, a *B* below 1/3 indicates substantial evidence for H0 over H1 (substantial only in the sense that the given evidence is just worth considering, Lee & Wagenmakers 2014). Therefore, a *B* between 1/3 and 3 indicates data insensitivity: H1 and H0 cannot be distinguished.

Below we test differences in the mean frequency of perspective switches according to groups and conditions by means of Poisson regressions on the raw count of switches per minute. To know the relative evidence for H1 versus H0, the predictions of H1 need to be specified. Crawford et al. (1993) found a mean difference of 6.06 switches per minute between highs and lows. Based on this, we can speculate that mediums should show a higher rate of perspective changes than lows, up to a maximum of 6. In order to have the same units between Crawford et al. 1993’s size effect and the coefficients of Poisson regressions, we took the log of the ratio between lows’ and mediums’ mean switch rate in Crawford et al. 1993’s, giving a value of 0.39. Then we modelled the alternative hypothesis H1 with a uniform distribution referred to as: *B*_*U*[0, 0.39]_ (Dienes 2014, 2015). In addition, regarding the potential differences in mean switch rate between highs and the other groups we might find, we modelled H1 with a half-normal distribution, written: *B*_*H*(0, 0.39)_.

### Regression

First, there was evidence in favour of H0 over the H1 for the difference between lows and mediums in Neutral Block (*ß* = 0.0280, SE = 0.0649, *z* = 0.43, *p* = 0.666, *B*_*U*[0, 0.39]_ = 0.15) as well as in Test Block in the Maintain (*ß* = 0.0254, SE = 0.0959, *z* = 0.265, *p* = 0.791, *B*_*U*[0, 0.39]_ = 0.25) and Switch (*ß* = −0.0195, SE = 0.0624, *z* = −0.31, *p* = 0.755, *B*_*U*[0, 0.39]_ = 0.26) condition. That is, these groups did not differ in their mean switch rate. Accordingly, in all the following analyses, we considered a factor group with two levels: highs on the one hand and lows + mediums on the other.

Then, we ran the Poisson regression with factors of block (neutral/test), instruction (switch/maintain) and group (lows + mediums versus highs), see Table 1 for results of the triple interaction. There was evidence for the three-way interaction (*ß* = 0.052, SE = 0.0107, *z* = 4.87, *p* = 1.10 × 10^−06^, *B*_*H*(0, 0.39)_ = 7966.90). We thus analysed the instruction by group two-way interactions within each block. For the Neutral Block, there was evidence for no instruction by group interaction (*ß* = −0.0134, SE = 0.0589, *z* = −0.23, *p* > 0.819, *B*_*H*(0, 0.39)_ = 0.18). In Test Block, there was marginal evidence for the two-way interaction (*ß* = −0.125, SE = 0.0581, *z* = −2.15, *p* < 0.0312, *B*_*H*(0, 0.39)_ = 2.77), to the effect that highs responded more to the instructions (delta switch rate = 12.4) than lows and mediums (delta = 3.61).

**Table 1:** Regression results

	Beta (*ß*)	Standard error	*z*	*p* value
Intercept	2.736	0.0539	50.69	2.00 × 10^−16^
Block	0.0516	0.0107	4.81	1.49 × 10^−6^
Instruction (switch/maintain)	0.140	0.0539	2.60	0.00927
Group	−0.117	0.0539	−2.18	0.0292
Block × instruction	−0.109	0.0107	−10.20	2.00 × 10^−16^
Block × group	−0.0540	0.0107	−5.03	4.81 × 10^−7^
Instruction × group	−0.0611	0.0538	−1.13	0.257
Block × group × instruction	0.0522	0.0107	4.87	1.10 × 10^−6^

Table shows results of the triple interaction for the Poisson regression

In addition, to test whether we replicated Crawford et al. (1993)’s results we tested the differences between groups in Neutral Block. Replicating Crawford et al. (1993)’s, there was indeed evidence for an effect of group (*ß* = −0.179, SE = 0.0589, *z* = −3.05, *p* < 0.005, *B*_*H*(0, 0.39)_ = 27.15), such that highs had a higher switch rate than lows and mediums. For comparison, we also compared groups in Test Block. In the Switch Condition, highs had a higher switch rate than lows + mediums (*ß* = 0.17, SE = 0.063, *z* = 2.68, *p* < 0.01, *B*_*H*(0, 0.39)_ = 11.00), while in the Maintain Condition, highs switched to the same extent as lows + mediums (11.8 and 12.9, respectively) (*ß* = −0.086, SE = 0.11, *z* = −0.81, *p* > 0.4, *B*_*H*(0, 0.39)_ = 0.16).

## Discussion

When no specific information is given to participants (Neutral Block), highs in comparison to lows and mediums show a higher rate of perspective changes with a Necker cube. In this respect, our results are in keeping with previous research having shown the same superiority effect of highs in comparison to lows when tested with a Necker cube or other bistable percepts (Crawford et al. 1993; Wallace 1986; Wallace & Garrett 1973; Wallace, Knight, & Garrett 1976; but see; Jamieson & Sheehan 2002). However, when specific information about their ability is provided to participants, highs in comparison to lows or mediums are much more affected by this specific information as shown by the triple interaction block by group by instruction.

Our results provide an alternative interpretation to the attentional account of groups switch rate differences (Crawford et al. 1993), that is in terms of behavioural strategy differences (Spanos 1986; cf Sheehan, Donovan, & Macleod 1988). We can speculate that highs reported more perspective changes in Neutral Block (and in previous studies reporting such effect) because they thought that this was the behaviour expected from them. Because they know they are highs and that the instruction was (both in our study and previous ones) to report the number of perspective changes, they might have inferred that they have to report a lot of changes and adopted strategies to fit their expectations. Therefore, changing their expectations would change their performances more than lows and mediums.

If our interpretation is correct, this means that highs interpreted the information given before Test Block (switch or maintain information) in a different way than lows and mediums did. While highs took the information at face value, and were motivated to respond to it, lows and mediums may have been less motivated. As a result, highs performed in Test Block as informed so that their performance deviated from neutral block as a function of the informational content of the condition they were assigned to (especially in the Maintain Condition). (As for the Switch Condition, highs may have reached ceiling in Neutral Block, preventing them to switch still more in Test Block). By contrast, the performance of lows and mediums was unchanged by the information given to them.

Another question that follow-up studies should address is whether highs adopted specific perceptual strategies in order to fit the content of the before-test-block information or simply increased (in the Switch Condition) and reduced (in the Maintain Condition) the rate of their response (compliance). The literature suggests that highs usually do their best to experience the content of suggestions delivered to them (e.g. Cojan, Waber, Schwartz, Rossier, Forster, & Vuilleumier 2009; Derbyshire, Whalley, & Oakley 2009; Kirsch, Silva, Carone, Johnston, & Simon 1989). The cognitive strategies that could maintain or switch a perspective include regulating attention to certain features to control bottom-up input (consider for example the longer switch times of meditators rather than non-meditators asked to maintain the perspective, Sauer, Lemke, Wittmann, Kohls, Mochty, & Walach 2012). Whether or not highs are better or worse at this is an open question, not settled by the greater switching of highs rather than lows in Neutral Block; our results motivate the claim that is a matter of strategy choice rather than ability.

The present results need to be tested for generalisability to differences between highs and lows in the context of other illusions. For example, highs in comparison to lows reported more changes in direction of autokinetic movement (Crawford et al. 1993; Wallace & Garrett 1973). As our results suggest, it might be that highs inferred that it was expected from them to perceive a lot of movements and so they used different strategies from lows to fit their experience to that implied by demand characteristics. The same explanation might be given to account for the higher frequency of apparent reversals in highs than in lows for rotary illusions (Wallace et al. 1976) or for the higher sensitivity of mediums and highs in comparison to lows to the Ponzo illusion (Miller 1975).

The results do not constitute a definitive argument against an account in terms of attentional abilities because the information we gave to participants before the test block was not explicit instructions to switch or maintain perspective. Therefore, we cannot fully exclude the possibility that highs (and lows and mediums for that matter) did not perform the best they could have been able to do. However, an alternative interpretation in terms of the implementation of different behavioural strategies between these populations remains a simple one. We showed that information about trait differences between highs and lows changed especially the behaviour of highs without us having to instruct them directly. Future studies with direct instructions for maintaining or switching perspective with motivation ratings will be necessary to favour one over another account.

## Electronic supplementary material

Below is the link to the electronic supplementary material.

Supplementary material 1 (CSV 198 KB)
